# Drug Combinations against *Borrelia burgdorferi* Persisters *In Vitro*: Eradication Achieved by Using Daptomycin, Cefoperazone and Doxycycline

**DOI:** 10.1371/journal.pone.0117207

**Published:** 2015-03-25

**Authors:** Jie Feng, Paul G. Auwaerter, Ying Zhang

**Affiliations:** 1 Department of Molecular Microbiology and Immunology, Bloomberg School of Public Health, Johns Hopkins University, Baltimore, Maryland, United States of America; 2 Fisher Center for Environmental Infectious Diseases, School of Medicine, Johns Hopkins University, Baltimore, Maryland, United States of America; University of North Dakota School of Medicine and Health Sciences, UNITED STATES

## Abstract

Although most Lyme disease patients can be cured with antibiotics doxycycline or amoxicillin using 2-4 week treatment durations, some patients suffer from persistent arthritis or post-treatment Lyme disease syndrome. Why these phenomena occur is unclear, but possibilities include host responses, antigenic debris, or *B. burgdorferi* organisms remaining despite antibiotic therapy. *In vitro*, *B. burgdorferi* developed increasing antibiotic tolerance as morphology changed from typical spirochetal form in log phase growth to variant round body and microcolony forms in stationary phase. *B. burgdorferi* appeared to have higher persister frequencies than *E. coli* as a control as measured by SYBR Green I/propidium iodide (PI) viability stain and microscope counting. To more effectively eradicate the different persister forms tolerant to doxycycline or amoxicillin, drug combinations were studied using previously identified drugs from an FDA-approved drug library with high activity against such persisters. Using a SYBR Green/PI viability assay, daptomycin-containing drug combinations were the most effective. Of studied drugs, daptomycin was the common element in the most active regimens when combined with doxycycline plus either beta-lactams (cefoperazone or carbenicillin) or an energy inhibitor (clofazimine). Daptomycin plus doxycycline and cefoperazone eradicated the most resistant microcolony form of *B. burgdorferi* persisters and did not yield viable spirochetes upon subculturing, suggesting durable killing that was not achieved by any other two or three drug combinations. These findings may have implications for improved treatment of Lyme disease, if persistent organisms or detritus are responsible for symptoms that do not resolve with conventional therapy. Further studies are needed to validate whether such combination antimicrobial approaches are useful in animal models and human infection.

## Introduction

Lyme disease, caused by the spirochetal bacterium *Borrelia burgdorferi*, is the leading tick-borne infection of humans in the US [1]. The clinical manifestations of early Lyme disease are most often characterized by an erythema migrans rash often accompanied by flu-like symptoms. An inflammatory arthritis or neurological dysfunction can be frequent sequelae of untreated infection. Although the majority of patients with Lyme disease can be cured with antibiotics doxycycline or amoxicillin used for 2–4 weeks durations, a subset of patients experience persistent symptoms despite antimicrobial therapy including fatigue, neurocognitive difficulties or musculoskeletal pains. When symptoms last longer than 6 months after antibiotic treatment, this has been proposed as a non-infectious, post-treatment Lyme disease syndrome (PTLDS) due to the inability to find viable, remaining organisms and lack of substantial efficacy with longer term monotherapy with ceftriaxone, doxycycline or amoxicillin [1–3].

It is unclear what mechanisms promulgate this condition in these patients. Concepts raised have included host responses, although slow or ineffective killing of *B. burgdorferi* persisters has been voiced as a possible explanation though evidence of viable organisms present in PTLDS is lacking [4]. While PTLDS has only subjective symptom complexes, about 10% of patients with late Lyme arthritis have objective, persistent joint swelling despite antibiotic therapy [5]. Though part of this response may include autoimmune mimicry induced by *B. burgdorferi* in certain hosts, an additional explanation rests on immunological responses driven by continued infection or presence of antigenic debris [6]. The question of whether *B. burgdorferi* might still persist in some patients with suboptimal immune clearance after antibiotic therapy and further evade host immune clearance has been raised by some but is controversial [7, 8].

In various animal models such as mice, dogs and monkeys, antibiotic therapy with doxycycline, ceftriaxone or tigecycline could not fully eradicate detection of *B. burgdorferi* including by xenodiagnosis though viable organisms could not be cultured in conventional culture medium [9–12]. Recently demonstrated, post-antibiotic persistence was present with resurgence of non-culturable *B. burgdorferi* DNA found in mice 12 months after antibiotic treatment [11]. These observations suggest some form of persistent *B. burgdorferi* that antibiotic dosings employed in these animal models are not able to completely eradicate, though antibiotic levels in these animals may have been inadequate. A number of prospective, randomized clinical studies have found neither significant beneficial effect of additional prolonged antibiotic therapy with conventionally employed antibiotic monotherapy nor evidence of continued presence of *B. burgdorferi* in patients with long-term symptoms [3, 13]. Intriguingly, a recent study in humans demonstrated the recovery of *B. burgdorferi* DNA by xenodiagnosis in a single patient with PTLDS despite antibiotic treatment [14]. One study did report some improvement in fatigue symptoms with prolonged intravenous administration with ceftriaxone, though ultimately not thought to be worth the risks to administer for this benefit alone [15]. Ceftriaxone has recently been shown to be more active against *B. burgdorferi* persisters experimentally than doxycycline or amoxicillin [16].

*B. burgdorferi* is capable of a complex life style *in vitro* characterized by multiple pleomorphic forms including spirochetal, spheroplast (or L-form), and cyst or round body (RB) form and microcolony forms [8, 17–20]. RB forms appear as coccoid atypical variants of *B. burgdorferi,* forming under experimental stress conditions such as starvation or antibiotic treatment in culture [17, 21]. These are relatively refractory to killing by many antibiotics including doxycycline and amoxicillin [16, 17], and can revert to classical helical spirochetal forms in fresh nonantibiotic-containing subculture [17, 22]. Atypical cystic or granular forms have been described in humans [19], but there is neither good evidence that such morphologic variants are common with human infection nor that additional antibiotics improves patients with persistent symptoms after initial treatment [23]. While frontline drugs doxycycline and amoxicillin kill replicating spirochetal form of *B. burgdorferi* quite effectively, they have little activity against non-replicating persisters or biofilm-like aggregates or microcolonies of *B. burgdorferi* enriched within stationary phase cultures [16, 24].

Taking advantage of a newly developed SYBR Green I/PI viability assay, we recently screened an FDA-approved drug library against stationary phase *B. burgdorferi* persisters and identified 27 drug candidates that individually have higher activity than the currently recommended Lyme antibiotics doxycycline or amoxicillin [16]. Among the top 27 confirmed drug candidates, daptomycin, clofazimine, carbomycin, sulfa drugs such as sulfamethoxazole, and certain cephalosporins such as cefoperazone showed higher activity against *B. burgdorferi* persisters [16]. Interestingly, some drug candidates such as daptomycin and clofazimine with the highest activity against non-growing persisters had poor activity against actively growing *B. burgdorferi* with high MICs, at 12.5–25 μg/ml and 6.25 μg/ml, respectively [16]. Although these drug candidates active against persisters may not have good activity when used alone due to their poor activity against growing *B. burgdorferi,* it raises the question whether they may be used with another antibiotic such as doxycycline that is effective at inhibiting or killing the growing forms of *B. burgdorferi.* Such combinations may yield more effective treatment of Lyme disease.

Experimentally, since a stationary phase culture contains mixed populations of growing and non-growing bacteria that have different morphological variants such as round bodies and microcolonies that are tolerant to antibiotics [16, 17, 24], it is most likely that a single drug may not effectively kill all bacterial populations including morphological variants. In this study, we evaluated a range of drug combinations with the aim to identify optimal drug combinations that are most effective at killing *B. burgdorferi* persisters.

## Results

### *B. burgdorferi* culture possesses different proportions of morphological variants including round body and microcolony forms as the culture ages

**Figure 1:**
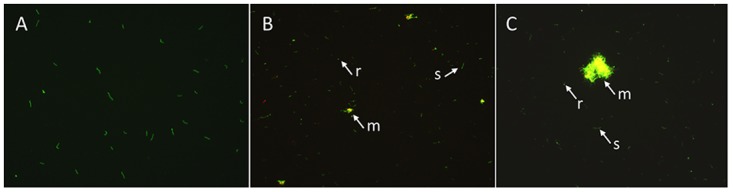
Representative images of 3 day old log phase (A), 7 day (B) and 10 day (C) old stationary phase *B. burgdorferi* cultures. The *B. burgdorferi* cultures of varying ages were stained with SYBR Green I/PI assay and observed under the microscope (400 × magnification). The arrows indicate the spirochete (s), round body (r), and microcolony (m) forms of *B. burgdorferi* in stationary phase cultures.

As shown in our previous study [16], the stationary phase culture was enriched with morphological variants such as round body form and biofilm-like aggregated microcolony form in increasing proportions in contrast to individual spirochetes found in log phase culture (Fig. 1). To more accurately assess the proportion of different morphological variant forms, we examined representative images of each sample taken from cultures of different ages to measure the percentage of different morphological forms of *B. burgdorferi* (Table 1). We found that the log phase (3 day old) *B. burgdorferi* culture consisted almost entirely of spirochetal form (96%), with few round body form (4%) and no aggregated microcolony form (Fig. 1A). In the 7 day old stationary phase culture of *B. burgdorferi*, there were 38% spirochetal form, 23% round body form, and 39% microcolony form (Fig. 1B). When *B. burgdorferi* stationary phase culture was cultured for 10 days, the percentage of the microcolony form increased to 64%, and the spirochetal form and the round body form were 20% and 16%, respectively (Fig. 1C).

**Table 1:** Varying degrees of susceptibility of different forms of *B. burgdorferi* to commonly used Lyme antibiotics.

	**Percentage of different forms of *B. burgdorferi*^a^**			**Percentage of residual viable cells^b, c^**			*E. coli* control^e^	
	**Spirochete**	**Round body form**	**Microcolony**	**Doxycycline**	**Amoxicillin**	**Ceftriaxone**	**Persister frequency^f^**	**Persister frequency^g^**
3 day log phase culture^d^	96%	4%	0%	8% (6.4%)	23% (9.6%)	6% (5.8%)	4.4%	0.9%
7 day stationary phase culture	38%	23%	39%	71% (24%)	80% (25%)	47% (16%)	-	-
10 day stationary phase culture	20%	16%	64%	80% (~25%)	83% (~27%)	70% (~25%)	-	-

a. Percentages of different forms of *B. burgdorferi* were calculated by measuring three representative microscope images with Image Pro-Plus software.

b. Percentages of residual viable *B. burgdorferi* relative to drug-free control after drug treatment were calculated according to the regression equation and ratio of Green/Red fluorescence obtained by SYBR Green I/PI assay as described [16]. The samples were treated with antibiotics for 7 days before viability was assessed by the SYBR Green I/PI assay.

c. Values in brackets indicate persister frequencies (percentage of live cells after antibiotic treatment). The number of *B. burgdorferi* assayed by epi-fluorescence microscope counting was calibrated using *E. coli* as a control.

d. The log phase culture was obtained by subculture of a stationary phase culture at 1:50 dilution for 3 days in BSK medium.

e. *E. coli* W3110 was 1:100 diluted and grown in LB broth. Three hour log phase *E. coli* culture (1.7 × 10^8^ cfu/ml) was added with 50 µg/ml amoxicillin, and incubated at 37°C with shaking for 3 hours followed by persister frequency determination by SYBR Green I/PI viability staining or CFU count.

f. Persister frequency calculated by epi-fluorescence microscope counting after SYBR Green I/PI viability staining.

g. Persister frequency calculated by standard plate colony count assay.

### Persister frequencies in log phase and stationary phase cultures

Because *B. burgdorferi* does not form colonies easily on agar plates, the conventional method to assay persister frequency after antibiotic exposure by calculating the percentage of bacteria killed by a bacteriocidal antibiotic cannot be applied to *B. burgdorferi.* Therefore, we assessed the frequency of *B. burgdorferi* persisters in log phase and stationary phase cultures using SYBR Green I/PI viability assay after exposure of the cultures to antimicrobials. We used *E. coli* culture as a control after exposure to antibiotics to validate the SYBR Green I/PI viability assay for persister frequency assessment. The persister frequency of the log phase *E. coli* culture with exposure to 50 µg/ml amoxicillin for 3 hours was 4.4% for the SYBR Green I/PI assay and 0.9% for the CFU assay (Table 1). Using SYBR Green I/PI assay the persister frequencies of *B. burgdorferi* ranged from 5–10% for log phase cultures, but ranged 16–27% in stationary phase cultures treated with ceftriaxone, doxycycline or amoxicillin (Table 1). Given the SYBR Green I/PI viability assay seemed to give about 5 fold (4.4%/0.9%) overestimation of the persister frequency over the CFU assay with the *E. coli* control, the real persister frequencies of *B. burgdorferi* are likely to be in the range of 1–2% for *B. burgdorferi* log phase cultures and 3–5.5% for stationary phase cultures.

### Microcolony form is more tolerant to antibiotics than free-living spirochetal and round body forms

Our previous study showed that the stationary phase *B. burgdorferi* was more resistant or tolerant to antibiotics than the log phase culture [16]. In view of the heterogeneity of the morphological variants of the stationary phase culture (Fig. 1B, 1C), we determined the susceptibility of different variant forms of *B. burgdorferi* to commonly employed antibiotics for Lyme disease (doxycycline, amoxicillin, and ceftriaxone) in a more quantitative manner. Interestingly, we found that different variant forms had differing susceptibilities to these antibiotics (Table 1). The log phase culture (3 day old) primarily consisting of spirochetal form was highly susceptible to these antibiotics, whereas the stationary phase (7 and 10 day old) cultures comprising mainly of round body and biofilm-like microcolony forms were less sensitive to these antibiotics, as shown by increasing proportion of viable cells remaining after the antibiotic exposure (Table 1).

**Figure 2:**
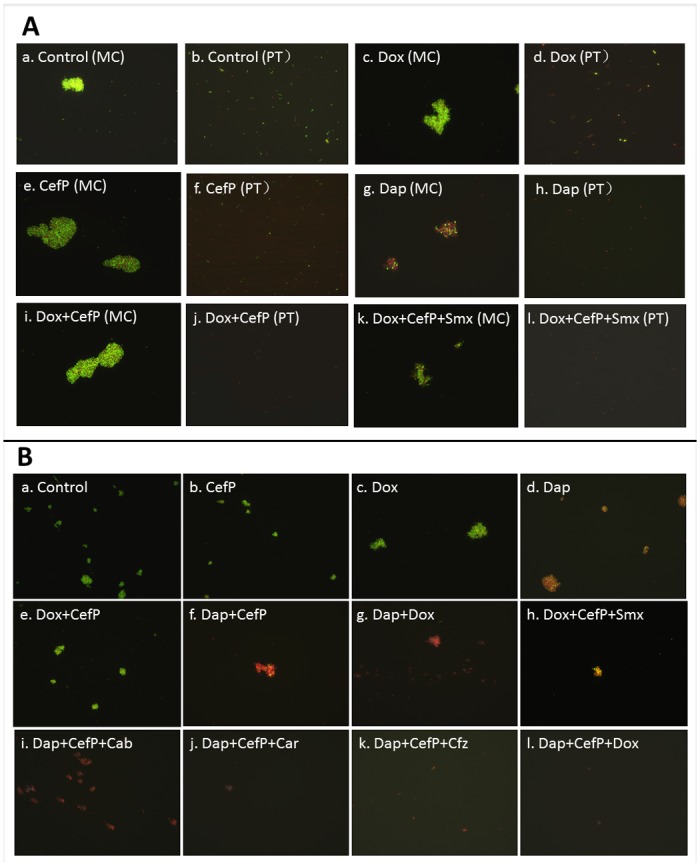
Effect of antibiotics alone and in combinations on aggregated microcolony form and planktonic forms of *B. burgdorferi*. Stationary phase *B. burgdorferi* culture (10 day old) was treated with 10 μg/ml drugs (labeled on the image) for 7 days followed by staining by SYBR Green I/PI assay. Green cells indicate live cells whereas red cells dead cells. (A) *B. burgdorferi* aggregated microcolony (MC) form was more resistant to different antibiotics or their combinations than planktonic form (round body and spirochetal form) (PT) as observed by fluorescence microscopy at 400 × magnification. (B) Susceptibility of *B. burgdorferi* microcolony form to antibiotics and antibiotic combinations was assessed by fluorescence microscopy at 200 × magnification. The luminance of individual RB is much weaker than that of microcolony, which makes the individual cells hard to be observed when the microcolonies were being examined. Abbreviation: Dox, doxycycline; CefP, cefoperazone; Cfz, clofazimine; Dap, daptomycin; Smx, sulfamethoxazole; Cab, carbencillin; Car, carbomycin.

When the 10 day old stationary phase culture, consisting of mixed populations of spirochetal form in minor portions and round body form and microcolony form in major proportions, was exposed to various antibiotics, the microcolony form was more tolerant to antibiotics than the free-living spirochetal form and the round body form. Daptomycin at 10 μg/ml, a drug with high activity against *B. burgdorferi* persisters [16] killed all planktonic forms (spirochetal and round body) of stationary phase cells (Fig. 2A, h) but could only partially kill the microcolony form of *B. burgdorferi* persisters as shown by the presence of significant numbers of red cells (dead cells) mixed with some green cells (viable cells) in the microcolony (Fig. 2A, g). The other persister active drug cefoperazone [16] had weaker activity than daptomycin since it had some activity for the planktonic form cells (52% cells were green cells) but little activity for the microcolony form of persisters where most of the microcolony cells remained as green (live) cells (Fig. 2A, e). In contrast, doxycycline had the least activity against stationary phase *B. burgdorferi* persisters where about 71% free-living planktonic cells including spirochetal form and round body form were not killed by doxycycline as shown by green (live) cells (Fig. 2A, Panel d) but the microcolony form was almost all live (Fig. 2A, Panel c). These findings suggest there is a differential tolerance or resistance in different variant forms of persisters *in vitro* (spirochetal form, round body form and microcolony in increasing order of resistance) to both current Lyme disease antibiotics and also even persister active antibiotics daptomycin and cefoperazone, with the microcolony form being the most tolerant to antibiotics.

### Effect of drug combinations on stationary phase *B. burgdorferi* persisters

Despite the powerful anti-persister activity of daptomycin and cefoperazone, they had limited activity to kill the most resistant microcolony form of persisters at 10 μg/ml (Fig. 2). These findings suggest that these FDA-approved persister drugs may have limited potential if used alone against *B. burgdorferi*. To identify more effective drug combinations that kill different variant forms of *B. burgdorferi* stationary phase persisters, we evaluated 81 drug combinations including FDA-approved drugs on a 10 day old *B. burgdorferi* culture enriched with microcolony and round body forms at 10 μg/ml of each individual drug (close to or lower than MIC). The results showed that some drug combinations were indeed much more effective than single drugs alone (Table 2). Among them, daptomycin highlighted itself as having the best activity against stationary phase *B. burgdorferi* persisters when combined with other drugs.

**Table 2:** Effect of drug combinations on stationary phase *B. burgdorferi* culture^a^.

**Live Cell %**	**C**	**Mcz**	**Pmb**	**Smx**	**Dap**	**Cfz^b^**	**Car**	**Van**	**Cab**	**Ofl**	**Clar**	**Tig**	**Hcq**	**Rif^b^**
C	87%	65%	77%^c^	82%^c^	52%	73%	65%	64%	67%	81%^c^	63%	67%	71%	82%^c^
Dox	72%	53%	64%	71%	32%	43%	59%	63%	64%	60%	60%	59%	68%	60%
Amo	75%^c^	51%	75%	68%	41%	56%	63%	-	-	-	-	-	-	60%
Ceftriaxone	68%	48%	64%	64%	41%	57%	63%	-	-	-	-	-	-	59%
Cefoperazone	64%	45%	64%	65%	41%	46%	60%	64%	63%	62%	63%	51%	53%	58%
Dox+CefP	59%	39%	59%	37%	**19%**	43%	55%	50%	59%	56%	55%	51%	48%	-
Dap	48%	37%	35%	**27%**	35%	**20%**	**26%**	**23%**	**20%**	**27%**	32%	**23%**	31%	-
Dap+Dox	34%	34%	33%	**24%**	32%	**20%**	**23%**	**25%**	**16%**	**20%**	31%	**21%**	**25%**	-

a. Ten day old stationary phase *B. burgdorferi* culture enriched with micro-colonies was treated with 10 µg/ml drugs alone or in different combinations (each drug is 10 µg/ml) for 7 days. The percentage of residual viable *B. burgdorferi* was calculated according to the regression equation and ratio of Green/Red fluorescence using the SYBR Green I/PI assay as described [16]. Direct microscopy counting was employed to verify the results of SYBR Green I/PI assay. The most effective drug combinations as reflected by residual viable cell percentages of less than 30% are shown in bold type. The best drug combinations without daptomycin are underlined. Abbreviations: Dox, doxycycline; Amo, amoxicillin; CefP, cefoperazone; Cfz, clofazimine; Mcz, miconazole; Pmb, polymyxin B; Dap, daptomycin; Smx, sulfamethoxazole; Cab, carbencillin; Car, carbomycin; Van, vancomycin; Ofl, ofloxacin; Clar, clarithromycin; Tig, tigecycline; Hcq, hydroxychloroquine; Rif, rifampin. “-” indicates not determined. C = drug-free control

b. To eliminate the influence of red color of antibiotics, fluorescence data was corrected using antibiotic control.

c. P-values of the standard *t*-test for the all treated group versus the drug-free control were less than 0.01 except the data marked with “c”.

Daptomycin (10 μg/ml) alone could not eliminate the microcolonies by itself (Fig. 2A, g), but daptomycin in combination with doxycycline or beta-lactams was very effective against *B. burgdorferi* planktonic persisters and also against microcolonies (Table 2, Fig. 2B). However, daptomycin in combination with doxycycline or cefoperazone produced better bacteriocidal activity against the microcolony form than either of these agents alone or drug combinations without daptomycin, such as doxycycline + cefoperazone or even doxycycline + cefoperazone + sulfamethoxazole as shown by more red cells (dead cells) being produced after treatment with daptomycin drug combinations (Fig. 2B, panel f, g, i, j, k and l). Nevertheless, daptomycin used as part of two drug combinations did not completely eradicate the more resistant microcolony form of persisters (Fig. 2B, panel f and g). Remarkably, daptomycin in a three drug combination with doxycycline and cefoperazone eradicated all microcolonies, whereas other daptomycin-containing three drug combinations using cefoperazone + either sulfamethoxazole or carbenicillin or carbomycin or clofazimine still had some traces of green/yellow cells remaining after treatment (Fig. 2B, panel h, i, j, k and l).

In addition to doxycycline and beta-lactams, some clinical drugs such as vancomycin, ofloxacin, clarithromycin, and hydroxychloroquine, which are not recommended for treating Lyme disease, also exhibited some weak activity on the 10 day old stationary phase *B. burgdorferi* culture, either alone or in combination with doxycycline and cefoperazone. Rifampin alone did not have significant activity for *B. burgdorferi* persisters but in combination with doxycycline, amoxicillin, ceftriaxone or cefoperazone had higher activity for *B. burgdorferi* persisters (Table 2). Among all the other non-daptomycin drug combinations, the only two drug combinations that are close to daptomycin drug combinations in killing *B. burgdorferi* persisters were Dox + either cefoperazone or miconazole or sulfamethoxazole (Table 2). In addition, clofazimine showed good activity against stationary phase *B. burgdorferi* persisters when combined with doxycycline and cefoperazone (Table 2). It is worth noting that the activity of carbenicillin, vancomycin, ofloxacin, clarithromycin, tigecycline, nisin, and hydroxychloroquine when combined with doxycycline only marginally enhanced doxycycline activity and their anti-persister activities were not as effective as when they were combined with daptomycin (Table 2).

### Subculture of antibiotic-treated *B. burgdorferi*

In our previous study, we found that daptomycin at 50 μM (equivalent to 81 μg/ml, a high dose to achieve in humans) had remarkable anti-persister activity that seemed to kill all *B. burgdorferi* persisters, as shown by all red cells stained by PI (Fig. 3D in Feng et al., 2014)[16]. To confirm that these red cells are indeed dead, we performed subculture test in fresh BSK-H medium and found that indeed these red cells treated with 50 μM daptomycin were dead as they failed to grow in the subculture test as shown by lack of any visible green spirochetes after 15 day subculture (data not shown). Having established the subculture test as a reliable assay for assessing the viability of antibiotic treated cells, we next proceeded to validating the above results obtained with select antibiotics or antibiotic combinations that produced the best bacteriocidal effects against persisters (Fig. 2).

**Figure 3:**
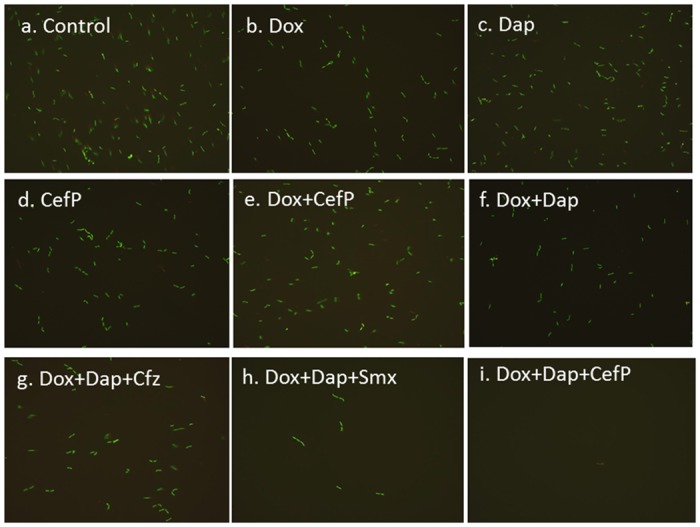
Subculture (15 days) of 10 day old *B. burgdorferi* stationary phase culture treated with different antibiotics alone or in combinations. Representative images were taken with fluorescence microscopy (400 × magnification) using SYBR Green I/PI staining. Only Dox+Dap+CefP completely killed all forms including the microcolony form of *B. burgdorferi* persisters as shown by lack of any viable green spirochetal form after 15 day subculture. Abbreviation: Dox, doxycycline; CefP, cefoperazone; Cfz, clofazimine; Dap, daptomycin; Smx, sulfamethoxazole.

To do this, we subjected a 7-day old stationary phase *B. burgdorferi* culture to exposure with select antibiotics and antibiotic combinations for 7 days, followed by subculture in fresh BSK-H medium for 7 days or 15 days. Microscope counting showed that drug-free controls and samples treated with single drug grew in the 7 day subculture. Samples treated with two drug combinations grew more slowly (Table 3). However, after 7 day subculture, all the three drug combinations, e.g., doxycycline+daptomycin+ either cefoperozone or sulfamethoxazole or clofazimine did not show any sign of growth as no visible spirochetal form was observed, whereas other drug combinations all had visible green spirochetes under the microscope. After 15 day subculture, there were about 6×10^6^ spirochetes in the control sample and about 5×10^6^ spirochetes in doxycycline or amoxicillin treated samples (Table 3). Interestingly, daptomycin alone, or two drug combinations doxycycline+cefoperazone and doxycycline+daptomycin, or even three drug combination doxycycline+daptomycin+clofazimine, could not sterilize the *B. burgdorferi* persisters, as they all had visible spirochetes growing after subculture (Fig. 3).

**Table 3:** Subculture tests to assess the viability of drug-treated stationary phase *B. burgdorferi.* ^a^

**Drugs^b^**	**Residual viable cells^c^**	**G/R ratio immediately after treatment^d^**	**Number of spirochetes after 7 day subculture^e^**	**Number of spirochetes after 15 day subculture^e^**
Control	82%	7.32	5×10^6^	6×10^6^
Dox	67%	6.71	9×10^5^	4×10^6^
Amoxicillin	80%	7.16	1×10^6^	6×10^6^
Dox+Dap+CefP	10%	5.32	<1×10^5^	<1×10^5^
Dox+Dap+Cfz	15%	4.96	<1×10^5^	6×10^6^
Dox+Dap+Smx	18%	5.85	<1×10^5^	2×10^6^
Dox+Dap	23%	6.03	3×10^5^	6×10^6^
Dox+CefP	56%	6.35	7.5×10^5^	4×10^6^
Dap	55%	5.85	3.1×10^6^	5×10^6^
CefP	61%	6.60	1.5×10^6^	5×10^6^

a. Seven day old *B. burgdorferi* culture (1×10^7^ spirochetes/ml) (500 μl) was treated with 10 μg/ml drugs alone or drug combinations for 7 days. Then, 50 μl of washed bacterial cells was subcultured in 1 ml fresh BSK-H medium for 7 days and 15 days.

b. Abbreviations: G/R ratio, Green/Red fluorescence ratio; Dox, doxycycline; CefP, cefoperazone; Cfz, clofazimine; Dap, daptomycin; Smx, sulfamethoxazole.

c. Residual viable *B. burgdorferi* was assayed by epi-fluorescence microscope counting.

d. Green/Red fluorescence ratios were obtained by microplate reader after SYBR Green I/PI staining. Each value is the mean of three replicates.

e. The number of spirochetes was evaluated by microscope counting.

However, doxycycline+daptomycin+sulfamethoxazole significantly reduced the number of spirochetes with very few spirochetes being visible after 15 day subculture (Fig. 3h). By far the best result was achieved with daptomycin in combination with doxycycline and cefoperazone, which killed all *B. burgdorferi* persisters with no viable bacteria observed (Fig. 3i). This is demonstrated by a decrease in the green/red fluorescence and lack of any viable green spirochetes, in contrast to samples treated with other drugs alone or drug combinations which all had higher Green/Red fluorescence and visible green spirochetal bacteria (Table 3, Fig. 3). Importantly, this drug combination could eliminate not only planktonic stationary phase *B. burgdorferi* persisters (spirochetal and round body forms) but also the more resistant biofilm-like microcolonies (Table 3, Fig. 3). Subculturing the sample treated with this drug combination showed no sign of any detectable organisms by microscopy (detection limit < 10^5^) even after 15 days of subculture (Table 3, Fig. 3i). These findings indicate that the microcolony structures are not eliminated by monotherapy (doxycycline, amoxicillin, persister active drugs), two drug combinations or even some three drug combinations, but could be eradicated by the drug combination of doxycycline, cefoperazone and daptomycin.

## Discussion

In this study, we conducted the first *in vitro* drug combination study using persister active drugs [16] in combination with the currently recommended Lyme antibiotics such as doxycycline or amoxicillin or other antibiotics to achieve more effective eradication of *B. burgdorferi* persisters. We found it is more effective to kill *B. burgdorferi* persisters by drug combination than single antibiotic, but bacteriocidal activity depended on the particular antibiotics used (Table 2). It is interesting to note that although persister active antibiotics such as the lipopeptide daptomycin and beta-lactam cefoperazone themselves were quite active against planktonic *B. burgdorferi* persisters (both spirochetal and round body forms), they were unable to eradicate the more resistant microcolony form when used alone or even in combination with some drugs (Fig. 2). Previous studies showed that tinidazole, metronidazole, and tigecycline were more active against *B. burgdorferi* round body and microcolonies than doxycycline and amoxicillin, but they could not completely kill the microcolonies even at high concentrations of antibiotics [24], indicating the limited activity of these individual antibiotics against *B. burgdorferi* persisters. Although tigecycline was the most active antibiotic against the round body form compared with tinidazole and metronidazole in that study [24], we found that by itself tigecycline was not very effective at killing the biofilm-like microcolonies (Table 2).

Remarkably, we found that daptomycin in combination with doxycycline and cefoperazone was able to completely eradicate the most resistant microcolonies (Fig. 2), and this was further confirmed by subculture studies which showed lack of any growth (Fig. 3). While various drug combinations showed improved activity against stationary phase *B. burgdorferi* persisters, daptomycin combinations had the best activity among drug combinations against persisters (Table 2). The only non-daptomycin regimens that were close to daptomycin combinations contained cefoperazone (Fig. 2, Table 2). Unexpectedly, other antibiotics such as sulfamethoxazole, clofazimine and miconazole also had more activity against stationary phase *B. burgdorferi* persisters in combination with doxycycline and cefoperazone. These drugs are not currently used as antibiotics for treatment of Lyme disease clinically [2, 25]. Although sulfa drugs are bacteriostatic when used alone for growing bacteria, they could kill non-growing round body or aggregated microcolony form of *B. burgdorferi* during long-term treatment. Clofazimine with high anti-persister activity improved the combination with daptomycin or daptomycin plus doxycycline (Table 2) which may be due to its multiple mechanisms of action including membrane destabilization, reactive oxygen species production, and inhibition of membrane energy metabolism in *M. tuberculosis* [26]. We also found that miconazole, an imidazole antifungal drug, had high activity against *B. burgdorferi* persisters when combined with doxycycline and cefoperazone (Table 2). Miconazole has been shown to alter the integrity of lipid membrane [27] and therefore may facilitate the penetration of other drugs such as doxycycline and cefoperazone for improved activity against *B. burgdorferi* persisters (Table 2).

The complete eradication of the *B. burgdorferi* biofilm-like microcolonies by the three drug combination of daptomycin+doxycycline+cefoperazone has not been achieved with any single, dual or even some three drug combinations in this study or any other previous studies. The mechanism by which this three drug combination was able to achieve this remarkable activity is worth commenting. Doxycycline and cefoperazone inhibits protein synthesis and cell wall peptidoglycan synthesis respectively [21]. Either may be needed to kill the growing forms present in the *B. burgdorferi* microcolonies or those occasionally revert to growing forms from microcolonies, but these drugs are less effective against the round body or microcolony persisters themselves [16, 17, 24]. This inability could be because of the reduced drug penetration into the microcolony structure, efflux mechanism [17, 28], or decreased protein or cell wall synthesis in persisters. The high efficacy of daptomycin against *B. burgdorferi* persisters could be due to its effect on membrane disruption or depolarization, resulting in a loss of membrane potential and inhibition of energy metabolism [16, 29], which is required for persister survival [30]. Prior studies have suggested that the combination of beta-lactams plus daptomycin increase effectiveness even with daptomycin resistant Gram-positive infections [31]. While drugs traditionally active against Gram-positive organisms are not thought to have activity against *B. burgdorferi*, *in vitro* studies have previously documented activity with drugs such as vancomycin [32, 33] but not teicoplanin or daptomycin, though this study was performed examining not persisters but log phase cultures. Though daptomycin is not used for Gram-negative pathogens, a drug such as colistin has been suggested to improve polyanionic lipopeptide activity due to outer membrane permeabilization [34]. Regardless, our studies suggest that combined use of these agents that kill or inhibit both growing organisms (doxycycline and cefoperazone) and non-replicating organisms (daptomycin and cefoperazone) may be important for good activity against the highly resistant microcolonies, which is consistent with the proposition to use drugs targeting both growing and non-growing microbial populations for improved treatment of persistent infections [30].

It is worth noting that short term incubation in subculture studies of antibiotic treated *B. burgdorferi* is not sufficient to assess the stable eradication of persisters. This is shown by 7 day subculture of *B. burgdorferi* persister cells treated with 3 drug combinations daptomycin+doxycycline+cefoperazone or Smx or Cfz, which all produced no detectable levels of any residual growth (Table 3). However, extended incubation to 15 days of subculture showed that only daptomycin, doxycycline and cefoperazone combination was able to completely eradicate biofilm-like microcolonies with no detectable spirochetes (Fig. 3i). These findings suggest that longer incubation to 15 days or more in post-antibiotic exposure may be needed to thoroughly assess the drug combinations that stably eradicate the persister forms without relapse. The subculture results do validate the SYBR Green I/PI viability assay and is a useful and more sensitive technique to assess the viability of *B. burgdorferi* persisters or microcolonies after drug treatment in identifying optimal drug combinations for killing more resistant persisters.

*B. burgdorferi* spirochetes could develop morphological variants as *in vitro* cultures age or are subjected to adverse conditions [16, 17, 20, 24, 35]. The proportions of these variants have not been well characterized over time in culture conditions. With careful measurement, the percentages of morphological variants were determined as they transitioned from spirochetes to progressively round body form to then microcolony form as log phase culture grew to stationary phase (7–10 days) (Fig. 1). Although previous studies reported the round body form or biofilm-like microcolony form is more resistant to antibiotics [16, 17, 24], their relative resistance was not fully studied. Here, we found a hierarchy or varying levels of stationary phase *B. burgdorferi* persisters in terms of their levels of persistence as the morphology of the variants changes from spirochetes, to round body, and to microcolony forms, with increasing antibiotic tolerance (Table 1). Future studies are needed to address the molecular basis of morphological transitions and their relationship to persistence in vivo.

The finding that persister frequencies are higher in stationary phase *B. burgdorferi* cultures than in log phase cultures is consistent with studies in other bacteria. However, the persister frequencies in *B. burgdorferi* log phase culture (5–10%) and stationary phase cultures (16–27%) determined by SYBR Green I/PI assay seem to be higher than those reported for *E. coli* [36]. CFU is the gold standard of current persister assays for bacteria that form colonies readily on agar plates. However, because *B. burgdorferi* does not form colonies easily on plates, especailly after antibiotic treatment, we have to rely on viability staining using microscope counting of green cells stained by viability dye SYBR Green even though CFU and viability by viability staining are two different measures of bacterial viability. To more accurately assess the percentage of *B. burgdorferi* persisters after antibiotic treatment, we used *E. coli* as a control to determine the correlation between the CFU assay and the SYBR Green assay. We found that the SYBR Green I/PI assay tended to overestimate the persister frequency by about 5 fold based on the *E. coli* data (4.4% persisters by SYBR Green viability assay versus 0.9% persisters by CFU assay) (Table 1). The overestimation of the persister frequency by SYBR Green I/PI assay may be due to some live persisters and/or injured cells that are unable to recover and form colonies on agar plates. Assuming the same correlation holds true for *B. burgdorferi,* the converted persister frequencies of 1–2% and 3–5% for *B. burgdorferi* log phase and stationary phase cultures would still suggest higher persister frequencies with *B. burgdorferi* than *E. coli,* which has 0.001% persisters in log phase and 1% in stationary phase [36]. In this study, the log phase *E. coli* had a considerably higher persister frequency of 0.9% by the CFU assay, and this could reflect differences in the higher inoculum and use of non-shaking condition, which could greatly increase the persister frequency. On the other hand, the higher persister frequencies for *B. burgdorferi* than *E. coli* could indicate that *B. burgdorferi* may form persisters more readily or reflect differences in the speed of growth of the organisms, the age of culture when antibiotic is added, and the dilution factor which affects the number of persisters carried over during the subculture. In addition, we found that the persister frequencies vary according to antibiotic exposure, with the more effective antibiotic ceftriaxone having a lower persister frequency than amoxicillin (Table 1), a finding that is consistent with previous studies [30, 37]. It remains to be determined if there are differences in persistence of *B. burgdorferi* strains and if the high persister frequencies in *B. burgdorferi* strains are associated with recalcitrance to antibiotic therapies.

In conclusion, we found there is a hierarchy of *B. burgdorferi* persisters with increasing antibiotic tolerance as the culture ages from log phase to stationary phase with morphological changes from spirochetal form to round body and microcolony forms. Importantly, we identified drug combinations that have high activity against *B. burgdorferi* persisters with daptomycin-containing combinations achieving the best activity. The most effective drug combination used daptomycin, cefoperazone and doxycycline which appeared to render resistant microcolony forms of *B. burgdorferi* unable to resuscitate viability upon subculture, a feature not previously described using any other antibiotic singly or in combinations. While important to state that the role of any persister organisms in human disease is far from elucidated, these findings may have implications for the treatment of certain Lyme disease patients with slow to resolve- or antibiotic-refractory arthritis or possibly stubborn ongoing symptoms. Direct extrapolation of these *in vitro* findings to human treatment would be unwise and premature. Future studies are needed to confirm whether such combination drug therapy yields benefit in animal models and possibly then in clinical studies.

## Materials and Methods

### Strain, media and culture

*Borrelia burgdorferi* B31 strain (ATCC 35210) was obtained from American Type Tissue Collection. Low passage (≤ 8) *B. burgdorferi* was cultured in BSK-H media (HiMedia Laboratories Pvt. Ltd.), with 6% rabbit serum (Sigma-Aldrich). All culture media were filter-sterilized by 0.2 μm filter. Cultures were incubated in sterile 50 ml conical tubes (BD Biosciences, California, USA) at 33°C without antibiotics. After incubation for 7–10 days stationary-phase *B. burgdorferi* cultures (100 μl, 1 × 10^6^ cells) were transferred into 96-well tissue culture microplate for evaluating effect of antibiotic treatment.

### Antibiotics

Doxycycline (Dox), amoxicillin (Amo), cefoperazone (CefP), clofazimine (Cfz), miconazole (Mcz), polymyxin B (Pmb), sulfamethoxazole (Smx), daptomycin (Dap), carbomycin (magnamycin A), vancomycin, nisin, carbencillin, ofloxacin, tigecycline, hydroxychloroquine, rifampin, and clarithromycin (Sigma-Aldrich, St. Louis, USA) were dissolved in suitable solvents [38] to obtain stock solutions. The antibiotic stocks were filter-sterilized by 0.2 μm filter except clofazimine which was dissolved in DSMO (dimethylsulfoxide) and not filtered. Then the stocks were stored at -20°C.

### Microscopy techniques

Specimens were examined on a Nikon Eclipse E800 microscope equipped with differential interference contrast (DIC) and epi-fluorescence illumination, and recorded with a Spot slider color camera. Cell proliferation assays were performed by direct counting using a bacterial counting chamber (Hausser Scientific Partnership, PA, USA) and DIC microscopy. SYBR Green I/PI assay was performed to assess the viability of *B. burgdorferi* as described [39]. The ratio of live (green) and dead (red) *B. burgdorferi* was calculated by counting these cells using a bacterial counting chamber and epi-fluorescence microscopy. The three representative images of every sample were captured for quantitative analysis. Image Pro-Plus software was applied to select green (including yellow) and red (including orange) areas of different morphological forms with calculation of the integrated fluorescence intensity (equal to area × average density or average intensity) of red and green portions as previously described [40].

### Antibiotic exposure assay

To qualitatively determine the effect of antibiotics, 10 μl of each compound from the pre-diluted plate or pre-diluted stock was added to stationary phase *B. burgdorferi* culture in the 96-well plate. The final volume per well was adjusted to 100 μl at a concentration of 10 μg/ml for each antibiotic. Plates were sealed and placed in 33°C incubator for 7 days. SYBR Green I/ PI viability assay was used to assess the live and dead cells after antibiotic exposure as described [16]. Briefly, 10 μl of SYBR Green I (10,000 × stock, Invitrogen) was mixed with 30 μl propidium iodide (PI, 20 mM) into 1.0 ml of sterile dH_2_O. Then, 10 μl of staining mixture was added to each well and mixed thoroughly. The plates were incubated at room temperature in the dark for 15 minutes followed by plate reading at excitation wavelength at 485 nm and the fluorescence intensity at 535 nm (green emission) and 635 nm (red emission) in microplate reader (HTS 7000 plus Bio Assay Reader, PerkinElmer Inc., USA). With least-square fitting analysis, the regression equation and regression curve of the relationship between percentage of live bacteria and green/red fluorescence ratios was obtained. The regression equation was used to calculate the percentage of live cells in each well of the 96-well plate. Each value is the mean of three replicates and the standard *t*-test was used for comparing the drug treated group and the drug-free control group.

For persister frequency assay, *E. coli* W3110 was diluted 1:100 in LB broth and shaken at 37°C. After 3-hour incubation, log phase culture was withdrawn, washed with LB broth, and spotted on LB agar plate to obtain the beginning colony counts. Meanwhile amoxicillin was added at a final concentration of 50 μg/ml, and shaken at 37°C. After 3-hour incubation, samples were withdrawn, washed with LB broth, and spotted on LB agar plate for colony counts.

### Subculture of antibiotic-treated *B. burgdorferi* to assess viability of the organisms

Seven day old *B. burgdorferi* culture (1×10^7^ spirochetes/ml) (500 μl) was treated with drugs or drug combinations in Eppendorf tubes. After incubation at 33°C for 7 days without shaking, the cells were collected by centrifugation and rinsed with 1 ml fresh BSK-H medium followed by resuspension in 500 μl fresh BSK-H medium without antibiotics. Then 50 μl of cell suspension was transferred to 1 ml fresh BSK-H medium for subculture at 33°C for 2 weeks. Cell proliferation was assessed using SYBR Green I/PI assay and bacterial counting chamber (Hausser Scientific Partnership, PA, USA) by microscopy as described above.
